# Oxidative Stress in Spinocerebellar Ataxia Type 3 and Its Attenuation by Herbal Remedies in Traditional Chinese Medicine: A Systematic Review

**DOI:** 10.3390/antiox13030375

**Published:** 2024-03-19

**Authors:** Nur Shahirah Mohd Hisam, Kah Hui Wong

**Affiliations:** Department of Anatomy, Faculty of Medicine, Universiti Malaya, Kuala Lumpur 50603, Malaysia; mic180032@siswa.um.edu.my

**Keywords:** spinocerebellar ataxia type 3, mutant ATXN3, oxidative stress, traditional Chinese medicine, antioxidant defense system

## Abstract

Spinocerebellar ataxia type 3 (SCA3) is an autosomal dominant neurodegenerative disorder that gives rise to motor incoordination and progressive functional disabilities. Although pharmacological interventions have revealed promising prospects in the management of SCA3, adverse effects may become unbearable. The use of herbal remedies in traditional Chinese medicine (TCM) may serve as potential alternative medicines to delay the progression of the disease. This systematic review is intended to identify, appraise, and summarize the findings of studies pertaining to the therapeutic roles of herbal remedies in TCM targeting oxidative stress in the management of SCA3. A literature search for relevant articles published from 1 January 2013 to 30 June 2023 in three databases, namely PubMed, Web of Science, and Scopus, was carried out according to the procedures of the Preferred Reporting Items for Systematic Reviews and Meta-Analyses (PRISMA). A total of ten preclinical studies met the inclusion criteria of the systematic review. We recognized the therapeutic potential of *Brassica napus*, *Codonopsis pilosula*, *Curcuma* sp., *Gardenia jasminoides*, *Gastrodia elata*, *Ginkgo biloba*, *Glycyrrhiza inflata*, *Hericium erinaceus*, *Hyptis* sp., *Paeonia lactiflora*, *Panax ginseng*, *Poria cocos*, *Pueraria lobata*, *Rehmannia glutinosa,* and *Scrophularia ningpoensis*. We identified the types of preclinical models expressing polyglutamine (polyQ) expanded mutant protein (mATXN3), inducers of oxidative stress that mimic the SCA3 pathogenesis, and effective doses of the herbal remedies. The modes of action contributing to the attenuation of oxidative stress are activation of antioxidant pathways, ubiquitin–proteasome system and autophagy, regulation of apoptosis, proinflammatory signaling pathway and chaperones, regulation of mitochondrial function and biogenesis, and restoration of neurotransmission and synaptic plasticity. In conclusion, herbal remedies in TCM may possibly delay the progression of SCA3, therefore providing justification for clinical trials.

## 1. Introduction

Spinocerebellar ataxia type 3 (SCA3), commonly known as Machado–Joseph disease, is an autosomal dominant neurodegenerative ataxia initiated by expanded cytosine–adenine–guanidine (CAG) triplet repeats in the coding region of *ATXN3*, specifically located at chromosome 14q32.1 [1]. Due to its pattern of inheritance, the mutant *ATXN3* (*mATXN3*) passes directly from one generation to the next, and each offspring who has a parent with the mutant gene has a 50% chance of getting the mutation [2]. The global prevalence of SCA is one to five cases in 100,000 individuals [3,4]. The highest prevalence has been found in Portugal [5], Norway [6], and Japan [7]. SCA3 dispersed almost exclusively by Portuguese emigration [8], thus explaining the high frequency of SCA3 in Portugal, especially in Flores Island of the Azores archipelago [9]. Furthermore, the disease is also common in Brazil, in which 69–92% of SCA families were diagnosed with SCA3, followed by 48–49% in China, 44% in the Netherlands, 42% in Germany, and 28–63% in Japan [10]. The onset of symptoms usually occurs between the ages of 30 and 50 and advances with age, with a mean survival period of 21 years following the onset [11].

Clinical manifestations of SCA3 include abnormal gait and stance, instability, impaired organization of posture and limbs, progressive dysarthria, dysphagia, and double vision. In addition, patients also suffer from involuntary weight loss, an inability to sleep, and mild cognitive impairments [12,13]. To date, there is no definitive cure to halt or delay its progression. Although the current pharmacological options could help in the management of SCA3, common adverse effects can include dizziness, nausea, and loss of appetite. The accumulating evidence indicates that mATXN3 protein aggregation or ATXN3 protein ablation results in mitochondrial dysfunction, decreased polynucleotide kinase 3′-phosphatase (PNKP) activity, and subsequently the accumulation of DNA strand breaks and oxidative stress-induced delayed repair of DNA strand breaks. As such, herbal remedies in TCM targeting the activation of antioxidant pathways and autophagy for the degradation of mATXN3 can be pursued for advancements in therapeutic interventions in TCM for SCA3. The systematic review is intended to critically discuss the current understanding of the therapeutic potential of herbal remedies in TCM, targeting oxidative stress in SCA3, published from 1 January 2013 to 30 June 2023. We examined multiple animal and cellular models of oxidative stress mimicking the pathogenesis of SCA3 and modes of action that contribute to the observed beneficial effects of SCA3. It is anticipated that these preclinical findings may open new opportunities and advancements in TCM by implementing herbal remedies in clinical studies.

## 2. The *ATXN3* Gene and ATXN3 Protein

The *ATXN3* encodes the polyubiquitin-binding protein named ataxin-3 (ATXN3). The ATXN3 protein regulates cytoskeletal dynamics [14] through interactions with tubulin and microtubule-associated protein 2 [15,16], acts as an ubiquitously-expressed deubiquitinating enzyme responsible for proteasome degradation of ubiquitinated proteins such as parkin [17], regulates transcription [18] through interaction with histones, activators, and repressors [19,20], maintains genome integrity through interaction with PNKP [21] and checkpoint kinase 1 (*CHEK1*) [22], and modulates ubiquitination of mediator of DNA damage checkpoint 1 (*MDC1*) [23] for DNA repair mechanisms [24].

The *ATXN3* contains CAG repeats within exon 10 of the *ATXN3* on chromosome 14q32.1 [25,26]. Normal alleles consist of 12 to 44 repeats, whereas alleles with 60 to 87 repeats are considered SCA3-causing alleles. Individuals with intermediate-size alleles of 45 to 59 repeats are not associated with a recognizable phenotype but are prone to instabilities of CAG repeats [27,28]. Similar to other polyQ diseases, the length of CAG repeats corresponds to the severity of the disease and, conversely, corresponds with the age of onset [29,30,31]. The phenomenon of anticipation has been reported in SCA3, referring to the tendency of expanded triplet repeats to become longer in the next generation [18], causing earlier onset and higher severity of the disease [13].

With the exception of neurons, in which the ATXN3 protein is predominantly a cytoplasmic protein, the accumulating evidence has revealed the localization of the protein in cytoplasm and nucleus of multiple types of cells, indicating other cell-specific factors being involved in the pathogenesis of SCA3. The exact mechanisms of misfolding and mATXN3 protein aggregation contributing to the clinical manifestations of SCA3 have not been fully clarified. Yang and Hu [32] reported that mATXN3 protein sequesters functional and interacting proteins implicated in protein quality control, causing malfunction of the proteins and interfering with protein homeostasis. As such, sequestration of PNKP, a protein of the DNA damage response (DDR), abolishes its function in the DDR cascade [33].

## 3. Oxidative Stress and Progression of SCA3

Redox homeostasis is critical for the appropriate functioning of cellular processes, including response against reactive oxygen species (ROS), signaling, regulation of thiols, oxidation–reduction reactions, and the elimination of xenobiotics. When the equilibrium is challenged, numerous signaling pathways are perturbed, leading to the onset of diseases. Oxidative stress is a consequence of a persistent imbalance between oxidants and antioxidants within the biological system of living organisms [34]. It is caused by the excessive synthesis of ROS, along with its primary types, namely superoxide anion (O_2_^•−^), hydrogen peroxide (H_2_O_2_), and hydroxyl radical (HO^•^), and an inability of biological systems to induce direct and indirect antioxidant mechanisms that are aimed at deactivating these harmful substances [35]. Among the undesirable effects are changes in the carbohydrate moieties that precipitate phosphodiester backbone incision in DNA and conformation changes in proteins, resulting in protein misfolding and aggregation [36]. Similarly, ROS attacks the membrane lipids, resulting in lipid peroxidation that forms a number of potentially toxic lipid aldehydes, including 4-hydroxy-trans-2-nonenal, acrolein, and malondialdehyde (MDA). Perhaps one of the longer-lasting effects of oxidative stress are mutations that could occur in the genome.

The surveillance role of ATXN3 in protein homeostasis has been widely documented, functioning in polyubiquitin binding and recruitment of polyubiquitinated substrates [13,37,38]. In SCA3, oxidative stress has been linked to a gradual decline in the activities of the ubiquitin–proteasome system (UPS), causing an accumulation of oxidatively modified proteins and transiently augmenting intracellular proteolysis. Indeed, interrupted functions of UPS are responsible for DNA repair, stress response, and cell proliferation, contributing to endoplasmic reticulum stress.

Additionally, an elevation of the intracellular level of ROS and reduced antioxidant capacity were detected in serum [39] and lymphoblastoid cells [40] derived from SCA3 patients. De Assis et al. [39] reported an increase in ROS and superoxide dismutase (SOD) levels and a decrease in glutathione peroxidase (GPx) levels in serum samples derived from early-to-moderate-stage SCA3 patients. Besides that, Araujo et al. [40] reported a decrease in superoxide dismutase 2 (SOD2) levels and enhanced ROS production in lymphoblastoid cells derived from SCA3 patients upon exposure to oxidative stress induced by H_2_O_2_. Yu et al. [41] also reported a decreased ratio of reduced glutathione (GSH) to oxidized glutathione (GSSG), catalase (CAT), glutathione reductase (GR), and SOD levels in SK-N-SH (human neuroblastoma) and COS-7 (African green monkey kidney fibroblast-like) cells transfected with full-length *ATXN3* with 78 CAG repeats (SK-N-SH-MJD78 and COS7-MJD78-GFP; GFP green fluorescent protein), lower than that of wild-type (WT) cells. There was also a decrease in mitochondrial DNA (mtDNA) copy numbers in blood samples from SCA3 patients, SK-N-SH-MJD78, and COS7-MJD78-GFP cells, suggesting that mtDNA defects may augment oxidative stress by reducing the expression of proteins facilitating the mitochondrial electron transport chain, leading to a vicious cycle of ROS and apoptosis.

The impairment of cellular defense mechanisms against oxidative stress could play a role in the pathogenesis of SCA3. In response to oxidative stress, various pathological conditions, including the dysregulation of signaling pathways and redox compartmentalization, cell apoptosis, and mitochondrial dysfunction, can occur [34].

### 3.1. Inactivation of Forkhead Box O4 Transcription Factor

The forkhead box transcription factor class O (FOXO) family represents a group of transcription factors that is crucial for the modulation of cell-cycle arrest, facilitating DNA repair before cell proliferation, and apoptotic function following oxidative stress [42,43]. Indeed, the activity of FOXO is regulated by post-translational modification, encompassing phosphorylation, acetylation, and ubiquitylation. The ATXN3 protein has been demonstrated to interact with forkhead box O4 (*FOXO4*), activating the *FOXO4*-dependent transcription of the *SOD2* gene.

Following oxidative stress, the ATXN3 protein and *FOXO4* translocate to the nucleus and bind to the *SOD2* gene promoter, leading to increased *SOD2* expression. Araujo et al. [40] found that the mATXN3 protein suppressed the activation of *FOXO4*-mediated *SOD2* expression and interfered with the binding of *FOXO4* to the *SOD2* gene promoter in pons tissue and lymphoblastic cells of SCA3 patients. This results in heightened ROS levels, causing damage to lipids, membranes, nucleic acids, proteins, and organelles, leading to cell death [40].

### 3.2. Inactivation of BCL2L1 Anti-Apoptotic Protein

Anti-apoptotic members of the B-cell lymphoma 2 (BCL2) family proteins are involved in the promotion or inhibition of apoptosis [44]. BCL2 like 1 (BCL2L1) is an anti-apoptotic protein that promotes cell survival [45], whereas BCL2 associated X, an apoptosis regulator (BAX) protein, is a pro-apoptotic protein that is essential for the permeabilization of the mitochondrial outer membrane [46]. Therefore, a balance between pro- and anti-apoptotic proteins is a critical point in the regulation of apoptosis.

Studies have reported that ATXN3 protein regulates the BCL2L1 and BAX protein complex, exerting protective effects against H_2_O_2_-induced oxidative stress in HeLa (human adenocarcinoma) and SH-SY5Y (human neuroblastoma) cells [47]. Overexpression of *BCL2L1* confers protection against oxidative stress, whereas knockdown of *BCL2L1* has been found to cause toxicity in the cells [47,48,49]. Further, heterodimerization of BCL2L1 protein with BAX protein has been observed to inhibit the activation of BAX in the attenuation of mitochondrial oxidative stress and cellular apoptosis [43,47].

On the other hand, the mATXN3 protein induces the mitochondrial apoptotic pathway through the downregulation of *BCL2L1* and upregulation of *BAX* messenger ribonucleic acid (mRNA) levels [50], resulting in the release of mitochondrial cytochrome c, somatic (CYCS), and the second mitochondria-derived activator of caspase (Smac), as well as the activation of caspase-9 [51,52]. Tsai et al. [53] reported that full-length expanded mATXN3 protein impaired BCL2L1 expression, resulting in an elevation of oxidative stress-induced cell death in SK-N-SH-MJD78 cells and SCA3 fibroblasts. Chou et al. [54] also reported a decrease in BCL2L1 and an increase in BAX protein expression in the pontine nuclei of 4-month-old SCA3 mice.

### 3.3. Inactivation of NFE2L2 Transcription Factor

The nuclear factor (erythroid-derived 2), like the bZIP transcription factor 2 (*NFE2L2*) gene, is a transcription factor that is critical for protecting cells against oxidative stress. The *NFE2L2*/antioxidant response element (ARE) system is responsible for regulating a battery of antioxidant enzymes in the detoxification and elimination of reactive oxidants, which is mainly achieved by conjugative reactions and the intensification of cellular antioxidant capacity [55].

Of the target genes regulated by *NFE2L2*, the reduced expression of phase II antioxidant enzymes, namely heme oxygenase 1 (*HMOX1*), NADPH oxidase 1 (*NADPH*), glutathione S-transferase pi 1 (*GSTP1*), glutamate-cysteine ligase catalytic subunit (*GCLC*), NAD(P)H quinone dehydrogenase 1 (*NQO1*), and phase II detoxifying enzymes, namely *GPx*, *GR*, glutamate-cysteine ligase, *HMOX1*, and *NADPH,* has been revealed to compromise the redox balance in SCA3. The mATXN3 protein also leads to impaired *NFE2L2* activation and decreased ARE binding activity [40], contributing to mitochondrial dysfunction and increased vulnerability to oxidative stress, resulting in the disruption of redox homeostasis.

In a study by Chang et al. [56], a decrease in *NFE2L2* expression and the knockdown of its transcriptional factor caused an increase in protein aggregation in human embryonic kidney (HEK-293) cells with inducible ATXN3/Q_75_-GFP expression. Similarly, a decrease in the nuclear NFE2L2 protein and *NFE2L2* transcriptional activity was also observed in Flp (flippase)-In SH-SY5Y cells with inducible N-terminal-truncated ATXN3/Q_14–75_-GFP expression [56] and SK-N-SH-MJD78 cells [57].

**Figure 1:**
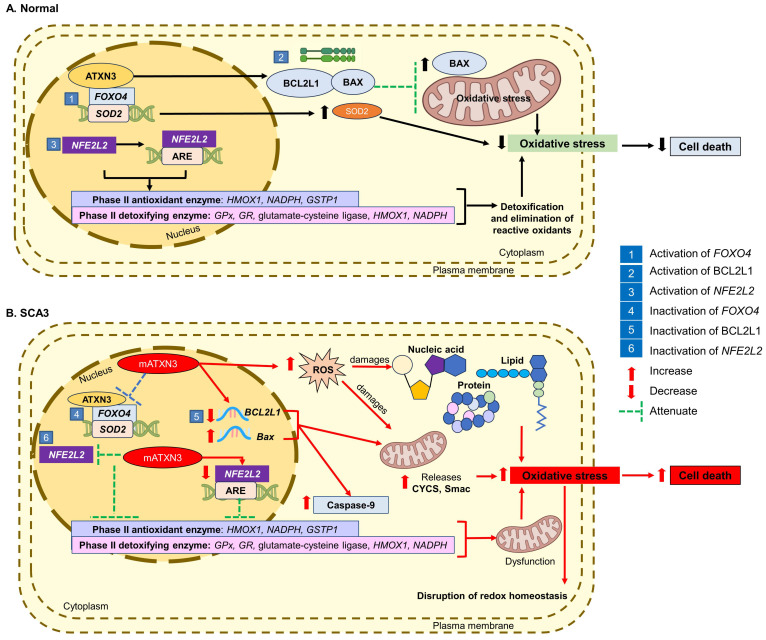
Oxidative stress in the pathogenesis of SCA3. (**A**) In normal condition, the activation of *FOXO4*, *BCL2L1*, and *NFE2L2* is essential regulators of cellular homeostasis. (**B**) In SCA3, mATXN3 protein inhibits he activation of *FOXO4*, *BCL2L1*, and *NFE2L2*, causing enhanced ROS production, mitochondrial dysfunction, and disruption of homeostasis, leading to cell death.

Figure 1 illustrates the cellular events of oxidative stress in the pathogenesis of SCA3.

## 4. Involvement of Mitochondrial Dysfunction in the Progression of SCA3

The ATXN3 protein is a ubiquitously expressed deubiquitinating enzyme that is responsible for the regulation of transcription, proteasome- and autophagy-mediated protein degradation pathways, DNA damage repair, and modulating cytoskeletal dynamics [18,58,59,60]. In polyQ disorders, including SCA3, diminished protection against free radicals caused by increased oxidative stress gives rise to mitochondrial dysfunction and cellular damage. Consistent with this observation, a cellular model overexpressing mATXN3 protein with 78 CAGs [41] and a transgenic mice model overexpressing mATXN3 protein with 98 to 104 CAGs [61] demonstrated reduced levels of antioxidant enzymes and increased mtDNA damage, indicating impaired mitochondrial function. The accumulation of mtDNA mutations and a reduction in the mtDNA copy number have been observed to disrupt mitochondrial bioenergetics, followed by downregulation of *SOD2* in the brainstem of SCA3 patients [40].

The full-length expanded ATXN3 protein has been reported to accelerate mitochondrial-mediated cell death and decrease the expression of BCL2 proteins, which can aggravate the mitochondrial dysfunction in SCA3, as demonstrated in SK-N-SH-MJD78 cells [53]. The BCL2 protein inhibits the mitochondrial permeability transition pore (mPTP) opening and release of apoptogenic proteins from the mitochondria, therefore preventing cell death induced by oxidative stress, ionizing radiation, and heat shock proteins (HSPs). As such, the findings by Yu et al. [41] and Kazachkova et al. [61] also showed that the mATXN3 protein can decrease mtDNA copy numbers and mitochondrial complex II, prompting reduced adenosine 5′-triphosphate (ATP) production, impaired calcium homeostasis, and depolarization of the mitochondria, leading to progressive oxidative stress.

Harmuth et al. [17] suggested the function of the ATXN3 protein as a deubiquitinating enzyme in the regulation and removal of ubiquitin from parkin prior to the degradation of impaired mitochondrial proteins, therefore inhibiting the self-ubiquitination of parkin in mitophagy. Parkin regulates the ubiquitination of numerous substrates at mitochondrial outer membrane proteins, such as voltage-dependent anion channel 1 (VDAC1) and mitofusins, thus driving the degradation of dysfunctional mitochondria through mitophagy mediated by the proteasome and the nucleoporin 62 (p62) protein. In events of elongated polyQ stretch, as in SCA3, the direct ubiquitination of VDAC1 by the mATXN3 protein is substantially enhanced, resulting in impaired polyubiquitination of VDAC1 by parkin, hindering mitophagy. This in turn activates apoptotic pathways through mitochondrial swelling, BAX/VDAC1 oligomerization, and the release of CYCS. Further, Harmuth et al. [17] reported a decrease in polyubiquitination of VDAC1 and failure of parkin recruitment to depolarized mitochondria in immortalized fibroblast cell lines derived from SCA3 patients, therefore proving the occurrence of parkin-independent mitophagy in the pathogenesis of the disease.

**Figure 2:**
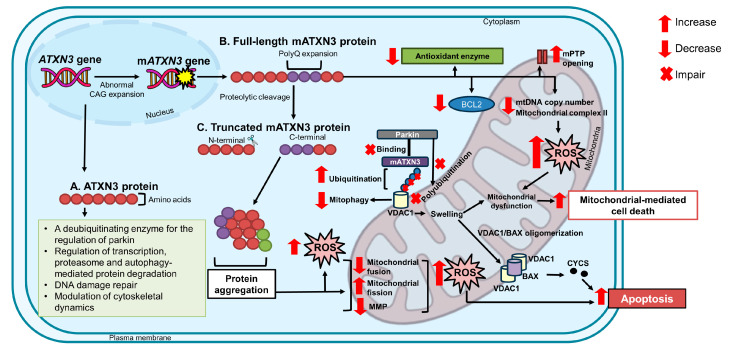
Mitochondrial dysfunction in the pathogenesis of SCA3. A: ATXN3 protein is necessary for the regulation of transcription, proteasome-, and autophagy-mediated protein degradation pathways, the removal of ubiquitin from parkin, DNA damage repair, and cytoskeletal dynamics. It functions as a deubiquitinating enzyme in the regulation of parkin prior to the degradation of impaired mitochondrial proteins. B: Full-length mATXN3 protein decreases the mtDNA number, disrupts the assembly of complex II, reduces the BCL2 protein, and prolongs mPTP opening, leading to mitochondrial dysfunction and mitochondrial-mediated cell death. C: Truncated mATXN3 protein enhances mitochondrial fission and decreases the mitochondrial fusion and MMP, leading to mitochondrial dysfunction, an accumulation of ROS, and eventually, apoptosis.

In addition, Hsu et al. [62] postulated that the mATXN3 protein undergoes proteolytic cleavage for the removal of the N-terminus, causing the progression of SCA3. Proteolytic cleavage of the mATXN3 protein has been observed to generate toxic fragments known as truncated C-terminal fragments, which appear as intranuclear aggregates in the brain samples of SCA3 patients. Generally, an overexpression of the truncated form of the mATXN3 protein results in impaired mitochondrial bioenergetic and protein conformational changes by enhancing mitochondrial fission and decreasing the expression of markers associated with mitochondrial fusion (mitofusin 1: *MFN1*; mitofusin 2: *MFN2*) and mitochondrial membrane potential (MMP), causing the accumulation of ROS and apoptosis. Mitochondrial dysfunction and neurodegeneration primarily affecting the cerebellar tissues were also observed in a transgenic mice model with the truncated mutant. Figure 2 outlines the events of impaired mitochondrial dynamics contributing to the pathogenesis of SCA3.

## 5. Impairment of DNA Damage Response in the Progression of SCA3

The accumulating evidence suggests that compromised DNA repair, or DDR, may contribute to the pathogenesis of SCA3. The DDR consists of a protein kinase cascade that facilitates in the detection and initiation of the DNA repair process. The process involves the phosphorylation of ataxia telangiectasia-mutated (ATM) and ATM-Rad3-related (ATR) apical kinases, followed by the activation of the mediator of *MDC1* and *PNKP*. The ATXN3 protein stabilizes *MDC1* and *PNKP* to stimulate the remodeling of damaged chromatin and recruitment of *PNKP*, ensuring DNA repair and cell survival [13]. The regulators of the cell cycle, namely *CHEK1* and tumor protein 53 (*TP53*), with the pro-apoptotic function are also activated by the phosphorylation of ATM and ATR apical kinases to induce cell-cycle arrest during DNA repair.

**Figure 3:**
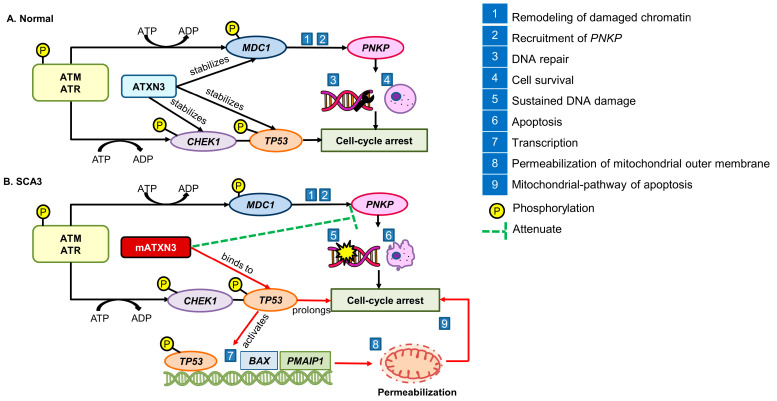
Alteration of DNA damage response in the pathogenesis of SCA3. (**A**) In normal conditions, the ATXN3 protein stabilizes *MDC1* to initiate remodeling of damaged chromatin and promote the recruitment of *PNKP*, *CHEK1*, and *TP53* to induce cell-cycle arrest, ensuring DNA repair and cell survival. (**B**) In SCA3, the mATXN3 protein inhibits *PNKP* and activates *TP53*-mediated neuronal death, causing mitochondrial pathway of apoptosis.

Conversely, the mATXN3 protein hinders the *PNKP*-dependent DNA repair process, causing prolonged cell-cycle arrest. Chou et al. [63] demonstrated that mATXN3 triggers *TP53*-mediated neuronal death in preclinical models by activating the transcriptional regulation of pro-apoptotic genes, namely *BAX* and phorbol-12-myristate-13-acetate-induced protein 1 (*PMAIP1*), leading to the mitochondrial pathway of apoptosis through permeabilization of the mitochondrial outer membrane. Sustained DNA damage and increased activation of pro-apoptotic pathways render the cells vulnerable to DNA damage [13]. Kazachkova et al. [61] and Chatterjee et al. [21] also observed compromised DDR that leads to the persistent accumulation of DNA damage in transgenic animal models and post-mortem tissues of SCA3 patients. Figure 3 outlines the events of alteration of DDR in the pathogenesis of SCA3.

## 6. Involvement of Antioxidant Defense Mechanisms in the Management of SCA3

Among the mechanisms responsible for governing redox homeostasis, enzymatic antioxidants, specifically the SOD, GPx, CAT, and non-enzymatic antioxidants, namely, vitamin A, C, E, lipoic acid, coenzyme Q10 (CoQ10), and glutathione, have been shown to mitigate the deleterious consequences of oxidative–nitrosative stress [64]. Antioxidants, either endogenously synthesized or obtained through the consumption of food and/or supplements [65,66], can scavenge ROS and reduce oxidative stress in SCA3, providing a translational framework for SCA3 research. For instance, CoQ_10_ has been reported to attenuate oxidative stress that causes cellular alterations, reduce mATXN3 protein aggregation in PC-12 cells transfected with full-length expanded ATXN3-Q84 [67], and restore the expression of antioxidant enzymes, namely NQO1, HMOX1, superoxide dismutase 1 (SOD1), SOD2, and CAT in SK-N-SH-MJD78 cells [68]. Interestingly, supplementation with CoQ_10_ also improved the scores for the Scale for Assessment and Rating of Ataxia (SARA) and the Unified Huntington’s Disease Rating Scale (UHDRS) in SCA3 patients [69,70].

Studies have revealed an imbalance between ROS production and antioxidant defense capacity in preclinical models of SCA3. Cellular abnormalities encompass transcriptional dysregulation, mitochondrial dysfunction, impaired proteasome and autophagy dynamics, and diminished axonal transport [71]. Although the precise mechanisms by which oxidative stress may accelerate the development and pathogenesis of SCA3 are not fully documented, Araujo et al. [40] suggested the protective role of ATXN3 protein in modulating the antioxidant capacity of *FOXO4*-dependent antioxidant stress responses. The process was interrupted following the expression of mATXN3 in pons tissue and lymphoblastic cells of SCA3 patients by suppressing the level of SOD2, indicating a decreased antioxidant capacity and an increased vulnerability toward oxidative stress that promotes neuronal death in SCA3. Further, the full-length mATXN3 protein has been observed to cause reduced levels of total glutathione and mitochondrial enzymatic activity in SK-N-SH-MJD78 cells [41]. The mutant protein is associated with decreased activities of CAT, GR, and SOD in SK-N-SH-MJD78 and COS7-MJD78-GFP cells, leading to the ineffective removal of oxides and H_2_O_2_, causing mitochondrial dysfunction. Therefore, therapeutic approaches aimed at restoring cellular antioxidant capacity may be beneficial in the management of SCA3 [40,72].

## 7. Traditional Chinese Medicine as an Alternative in the Management of SCA3

TCM emerged as a medical practice in ancient China more than 3000 years ago. Its theoretical foundation is derived from the ideas of qi (vital energy), yin-yang (mutually interconnected forces), and wu xing (five phases), which include water, fire, earth, metal, and wood [73]. Body–mind–spirit practices (qi gong and tai chi), moxibustion, acupuncture, and herbal formulation are the most often employed therapeutic methods in TCM for maintaining balance, improving circulation, and boosting energy [74].

Evidence-based research in the clinical pharmacology of TCM sets the therapeutic basis for understanding disease and clinical prescription. In Shen Nong Ben Cao Jing (Divine Farmer’s Classic of Materia Medica), Chinese medicines were classified according to the concept of semi-supervised incremental clustering. The text is divided into three scrolls documenting 120 ‘upper’ herbs to extend the lifespan, 120 ‘middle’ herbs to prevent illness, and 125 ‘lower’ herbs to treat illness [75]. In line with this, herb-, plant- and mushroom-based therapeutic agents have been utilized by TCM practitioners. The efficacy of TCM is attributed to the synergistic effects of multi-herb formulations in hitting several targets, therefore advocating the combination of herbs in prescriptions to achieve maximum effects [76].

The past decade has witnessed a huge growth in the popularity of TCM and its compounds due to highly distinctive patterns of association between phytochemical classes and molecular target profiles. Recent research findings revealed the beneficial effects of TCM formulation and its associated representative compounds of alkaloids, bibenzyls, flavonoids, phenylpropanoids, quinones, and terpenoids in the management of neurodegenerative diseases [77,78,79,80] that are considered multifactorial conformational diseases. The formulation has been found to exert neuroprotective effects against depression [81], epilepsy [82], hypoxic-ischemic brain injury [83], and traumatic brain injury [84], as well as modulate the antioxidant defense system in the treatment of spinal cord injury [85]. Nevertheless, these compounds have not been evaluated and validated in clinical trials involving SCA3 patients. In a phase 2, randomized, double-blind, placebo-controlled, and crossover interventional study conducted at Changhua Christian Hospital in Taiwan, an administration of undeclared composition of TCM formulation for 36 weeks has been suggested to modulate insulin-like growth factor-1 (IGF-1) signaling systems among the young and elderly subjects diagnosed with SCA3. Indeed, IGF-1 has been shown to trigger signaling pathways and improve mitochondrial function, as well as glycolysis, in the restoration of cellular functions [86].

Protein misfolding, polymerization, and aggregation in the brain tissues are the hallmark events implicated in diverse neurodegenerative diseases. As the core intracellular machinery for degrading aggregated proteins and damaged organelles, autophagy has been found to eliminate aberrant protein aggregates in the brain tissues of patients diagnosed with Alzheimer’s disease (AD), Parkinson’s disease (PD), Huntington’s disease, and amyotrophic lateral sclerosis [87]. The accumulation of abnormal protein further overwhelms the autophagosome–lysosome pathway. The failure of accumulated modified proteins to be destroyed in UPS triggers a vicious cycle of ROS formation, mtDNA damage, and mitochondrial dysfunction. The TCM formulation that regulates autophagy through the phosphoinositide-3-kinase (PI3K)/protein kinase B (AKT)/mechanistic target of the rapamycin kinase (mTOR) pathway, beclin-1/BCL2 complex, 5′-adenosine monophosphate (AMP)-activated protein kinase (AMPK)/unc-51-like autophagy activating kinase (ULK)/mTOR pathway, transcription factor EB (*TFEB*), and PTEN-induced kinase 1 (PINK1)/parkin pathway [88] has gained increasing popularity in the drug discovery field. Taken together, the degradation of mATXN3 through autophagy and stimulation of the antioxidant defense system contributing to neuroprotective effects can be targeted for the advancements in therapeutic interventions of TCM for SCA3.

## 8. Materials and Methods

### 8.1. Registration

The protocol has been registered with the International Prospective Register of Systematic Reviews (PROSPERO) [Centre for Reviews and Dissemination (CRD), University of York, York, United Kingdom]; registration number: CRD42023396890.

### 8.2. Search Strategy

We conducted a search using Boolean terminology in three electronic databases (Web of Science, PubMed and Scopus) for full-length peer-reviewed articles published from 1 January 2013 to 30 June 2023. The Boolean search strings are as follows: (((((oxidative stress) OR (oxidative damage)) OR (oxidative insult)) OR (oxidative injury)) AND ((((SCA3) OR (spinocerebellar ataxia type 3)) OR (Machado-Joseph disease)) OR (Machado Joseph Disease))) AND ((((((((((((((natural remedy) OR (herbs)) OR (spices)) OR (mushroom)) OR (plants)) OR (fungi)) OR (algae)) OR (traditional medicine)) OR (traditional Chinese medicine)) OR (decoction)) OR (secondary metabolites)) OR (vitamin)) OR (polysaccharide)) OR (food)).

### 8.3. Eligibility Criteria

Studies were included if they fulfilled the following criteria: (i) preclinical studies; (ii) SCA3 being the primary disorder; (iii) English-language article; and (iv) full text available. Studies were excluded if they did not meet the following criteria: (i) non-motor symptoms of SCA3; (ii) drug-induced SCA3; (iii) review articles; (iv) meta-analysis; (v) conference abstracts; (vi) proceedings; (vi) non-English language article; and (vii) full text unavailable.

### 8.4. Data Extraction and Analysis

Data extraction and collection were performed in accordance with the Preferred Reporting Items for Systematic Review and Meta-Analyses (PRISMA) [89]. Duplicates were removed, and titles and abstracts were carefully screened and selected according to the inclusion criteria. Any disagreements during the process of screening were resolved through discussion with a third reviewer.

## 9. Results

**Figure 4:**
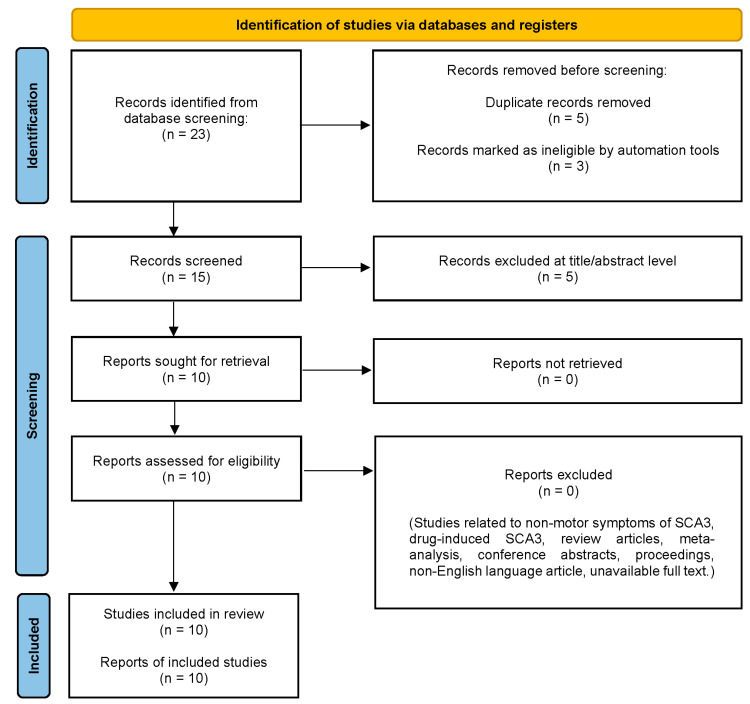
PRISMA flow chart for the identification and selection of relevant studies.

The literature search yielded 23 potentially relevant items. After removing five duplicate records and the other three records that were marked as unqualified by automation tools, the remaining fifteen items were further examined for titles and abstracts. Consequently, a total of 10 articles that met the inclusion criteria were retrieved for further assessment. Figure 4 shows the PRISMA flow chart for the identification of the relevant studies.

## 10. Discussion

**Figure 5:**
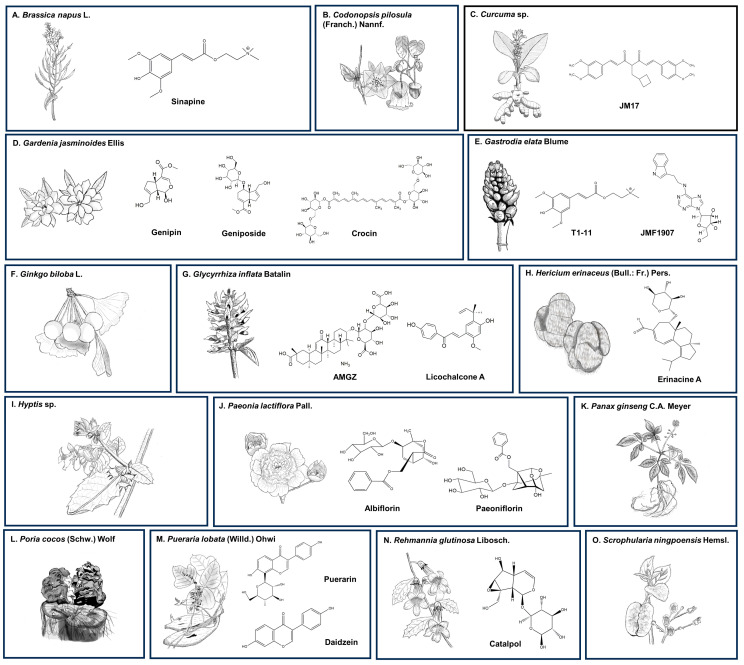
The herbal remedies in TCM and their corresponding bioactive compounds in the management of SCA3.

Figure 5 shows the herbal remedies in TCM and their corresponding bioactive compounds that exhibit therapeutic effects against SCA3. The in vitro studies were mainly conducted on transfected cells designed to drive the expression of mATXN3 for a better understanding of the impact of polyQ expansion on cell survival [59], dysregulation of autophagy [90], and pathogenesis [91] of SCA3. The studies employed human neuroblastoma SK-N-SH cells transfected with full-length *ATXN3* with 78 CAG repeats (SK-N-SH-MJD78), human embryonic kidney 293 (HEK-293), or SH-SY5Y cells with inducible ATXN3/Q_75_-GFP expression, Flp-In SH-SY5Y cells with inducible N-terminal-truncated ATXN3/Q_14–75_-GFP expression, induced pluripotent stem cells (iPSCs), and GFPu reporter cells. On the other hand, animal studies were conducted in transgenic *Caenorhabditis elegans* expressing mATXN3, transgenic mice expressing mATXN3-Q79HA, tert-butyl hydroperoxide (tBH)-induced oxidative stress in ELAV-SCA3tr-Q78 *Drosophila melanogaster,* and juglone-induced oxidative stress in WT *C. elegans.* Here, we review the in vitro and in vivo studies for assessing the therapeutic potential of herbal remedies in TCM in the management of SCA3. Table 1 shows the details of these studies, comprising the model, dose, finding, and mode of action.

### 10.1. Brassica napus L.

*Brassica napus* L., commonly known as Ou Zhou You Cai and rapeseed, is a bright-yellow flower belonging to the family Brassicaceae [99]. In the last few decades, rapeseed has been cultivated on a large scale due to its oil-rich seed, a key ingredient in producing edible rapeseed oil, animal feed, and biofuel [100]. Rapeseed pomace (RSP) is a by-product of rapeseed oil production that has traditionally been used to feed livestock [101]. At present, RSP is a source of nutraceuticals owing to its high content of secondary metabolites such as carotenoids, phospholipids, polyphenols (sinapic acid and its derivatives), sterols, tannins, and tocopherols [102].

Pohl et al. [92] highlighted the potential use of RSP ethanol extract in the mitigation of oxidative stress in an in vivo model of transgenic *C. elegans* expressing mATXN3 proteins. The administration of 0.1–5 mg/mL of ethanol extract activated transcriptional regulation of glutathione S-transferase alpha 4 (*GSTA4*) and superoxide dismutase 3 (*SOD3*), leading to restoration of motor function and recovery of the motility defect. The observed effects were comparable to those of WT, whereas knockdown of *GSTA4* diminished motor function in a motility-based screening. Interestingly, although sinapine, a secondary metabolite derived from the extract, also contributed to enhanced motor function, it is less effective than that of the whole extract, suggesting a synergistic therapeutic action of multi-constituents of ethanol extract.

### 10.2. Curcuma sp.

The *Curcuma* genus belongs to the family Zingiberaceae [68], which is widely distributed in tropical regions including Southeast Asia and India. Curcumin, a yellow pigment and an active constituent of *Curcuma longa* L. (Jiang Huang), commonly known as turmeric, possesses anti-inflammatory, anticarcinogenic, anti-helminth, cardioprotective activities, and neuroprotective benefits [103,104,105].

Wu et al. [68] demonstrated the therapeutic potential of JM17, a curcumin analog in the mitigation of oxidative stress in an in vitro model of SK-N-SH-MJD78 cells. The administration of 0.3–5 µM of JM17 rescued impaired mitochondrial metabolism associated with respiratory chain dysfunction by activating *NFE2L2* signaling monitored by cellular oxygen consumption rates. The observed effects were comparable to that of omaveloxolone (RTA408) and dimethyl fumarate (DMF), the activators of *NFE2L2.* Besides that, the administration of 0.3 or 1 µM of JM17 reduced mATXN3 protein expression and aggregation without a notable improvement in ATXN3 mRNA expression. Further, the treatment also promoted the degradation of mATXN3 through autophagy involving the upregulation of p62 and microtubule-associated protein light chain 3 (LC3)-II protein expression, significantly decreased the elevated intracellular level of ROS compared to that of RTA408 and DMF and increased the protein expression of NQO1, HMOX1, SOD1, and SOD2, and levels of CAT, total glutathione, and GSH through the upregulation of *NFE2L2* transcriptional activity. The findings suggested that JM17 ameliorated oxidative stress in SCA3 by improving mitochondrial function and reducing polyglutamine neurotoxicity.

### 10.3. Gardenia jasminoides Ellis

*Gardenia jasminoides* Ellis, commonly known as Zhi Zi, is an evergreen flower-bearing plant belonging to the family Rubiaceae. It is widely distributed in Southeast Asia. A total of 162 compounds have been isolated from *G. jasminoides* [106], of which geniposide, an iridoid glycoside, has been found to possess anti-inflammatory [107], antidepressant [108], antioxidant [109], antidiabetic [110], and neuroprotective properties [111], justifying the use of *G. jasminoides* in herbal formulations to treat fever, headache, inflammation, jaundice, edema, and hepatic disorders.

Chang et al. [56] revealed the therapeutic potential of *G. jasminoides* in the mitigation of oxidative stress in in vitro models of HEK-293 cells with inducible ATXN3/Q_75_-GFP expression and Flp-In SH-SY5Y cells with inducible N-terminal-truncated ATXN3/Q_14–75_-GFP expression activated by doxycycline and oxaliplatin. The administration of 1–10 µg/mL of aqueous extract, 50–500 of nM genipin, 500 of nM of geniposide, and 100 nM of crocin decreased the ATXN3/Q_75_ protein aggregation through an upregulation of NFE2L2 protein expression and its downstream targets of phase II antioxidant enzymes, namely NQO1, GCLC, and GSTP1 protein expression. The beneficial roles of *G. jasminoides* in exerting protective effects against oxidative stress were associated with decreased caspase-3 activity and ROS levels. Moreover, a derivative of HEK-293 cells, expressing a mutant SV40 large T antigen and ATXN3/Q_75_ showed a decrease in mATXN3 protein aggregation following transfection with the transcription factor *NFE2L2*. In contrast, a knockout of *NFE2L2* in HEK-293 cells has been found to promote aggregation formation.

### 10.4. Gastrodia elata Blume

*Gastrodia elata* Blume, commonly known as Tian Ma, is a perennial herb belonging to the family Orchidaceae [112]. It is distributed in the mountain regions of China, Korea, Japan, and India [113]. In the last few decades, approximately 80 compounds have been isolated and identified from *G. elata*, including gastrodin, organic acids, phenolics, and sterols [114], suggesting its complementary role in TCM to treat tetanus, neurasthenia [113], headache, dizziness [115], and neurological diseases such as stroke [116], epilepsy [117], and vascular dementia [118].

Chou et al. [54] reported the therapeutic potential of T1-11 [N6-(4-hydroxybenzyl) adenosine] derived from *G. elata* and JMF1907 (N6-(3-indolylethyl) adenosine), a synthetic analogue of T1-11 in the mitigation of oxidative stress in an in vivo model of SCA3 transgenic mice expressing hemagglutinin (HA)-tagged polyglutamine-expanded ataxin-3-Q79 (ATXN-3-Q79HA). The compounds reversed mATXN3-Q79HA-induced upregulation of pro-apoptotic BAX protein and downregulation of anti-apoptotic BCL2L1 protein and suppressed the formation of caspase-3 and caspase-9 in pontine nuclei following oral administration of 50 mg/kg for 3 months. Moreover, T1-11 or JMF1907 also enhanced the proteasome and chymotrypsin-like proteasome activities and decreased the protein level of mATXN3-Q79HA in the cerebellum and pontine nuclei assessed by fluorescent probe 7-amino-4-methylcoumarin and immunoblotting assay, respectively, without a significant change in the mRNA expression of mATXN3-Q79HA in pontine nuclei. Additionally, Chou et al. [54] also observed an improvement in the balance and coordination on the rotarod performance test, suggesting that the compounds may suppress mATXN3-Q79HA-induced damage in pontine nuclei and dysregulation of cerebellar transcription. Interestingly, the compounds also restored the downregulation of mRNA expression of myosin VA (*MYO5A*), synaptotagmin 1 (*SYT1*), calcineurin subunit B (*CN-B*), inositol 1,4,5-trisphosphate receptor type 1 (*ITPR1*), and phospholipase C beta 4 (*PLCB4*), re-establishing neurotransmission and synaptic plasticity in the cerebellar tissues.

### 10.5. Glycyrrhiza inflata Batalin

*Glycyrrhiza* sp., commonly known as licorice, belongs to the family of Fabaceae (also known as Leguminosae) and is native to central Asia, Iraq, Mongolia, and northwestern China. A total of 20 triterpenoids and 300 flavonoids have been isolated from *G. inflata* (Gan Cao). Among the compounds identified, glycyrrhizin, 18β-glycyrrhetinic acid, liquiritigenin, licochalcone A, licochalcone E, and glabridin have been reported to exert anti-inflammatory, antioxidant, and neuroprotective effects [119].

Chen et al. [93] demonstrated protective effects of *G. inflata* in the mitigation of oxidative stress in in vitro models of HEK-293 cells with inducible ATXN3/Q_75_-GFP expression and Flp-In SH-SY5Y cells with inducible N-terminal-truncated ATXN3/Q_14–75_-GFP expression activated by doxycycline and oxaliplatin. The administration of 5–5000 µg/mL of aqueous extract successfully decreased mATXN3 protein aggregation owing to the upregulation of the peroxisome proliferative activated receptor, gamma, coactivator 1 (*PPARGC1A*) mRNA expression, and its downstream target genes, namely *SOD2* and *CYCS,* along with *NFE2L2* mRNA expression and its downstream gene expression, namely *HMOX1*, *NQO1*, *GCLC*, and *GSTP1*. The protein encoded by *PPARGC1A* regulates mitochondrial biogenesis and genes of antioxidant response, whereas *NFE2L2* promotes the upregulation of ARE-dependent gene expression following oxidative stress [114]. Further, 50 µg/mL of the aqueous extract, 1 µM of ammonium glycyrrhizinate (AMGZ), and 10 nM of licochalcone A attenuated oxidative stress in HEK-293 cells with inducible ATXN3/Q_75_-GFP expression by increasing the ratio of GSH to GSSG, decreasing the ROS level and caspase-3 activity, and scavenging of free radicals (extract and licochalcone A).

### 10.6. Hericium erinaceus (Bull.: Fr.) Pers.

*Hericium erinaceus* (Bull.: Fr.) Pers. commonly known as Hou Tou Gu, lion’s mane mushroom, pom pom mushroom, and yamabushitake, belongs to the division of Basidiomycota and is distributed in Asia, Europe, and North America [78]. It has been used in TCM practices for thousands of years to alleviate gastrointestinal discomfort. Therapeutic potentials of *H. erinaceus* have been demonstrated in preclinical studies of neuroregeneration [120,121,122], depressive-like disorder [78,123,124], cerebellar ataxia [125,126,127], PD [128], AD [129,130], and traumatic brain injury [131]. A total of 80 secondary metabolites, namely hericenones, erinacines [132,133], erinapyrones [134,135], sterols, fatty acids, and fumitremorgin C [136] have been isolated from the fruiting bodies and mycelium of mushrooms.

Wu et al. [94] observed the protective effects of *H. erinaceus* in the mitigation of oxidative stress in an in vitro model of tBH-induced neurotoxicity in SK-N-SH-MJD78 cells. The administration of 5 µg/mL of *H. erinaceus* mycelial extract containing a substantial amount of erinacine A improved the viability, attenuated apoptosis and mATXN3 protein aggregation, and regulated the expression of polyQ-expanded mATXN3 and heat shock 27kD protein 1 (HSP27) proteins. The effects were achieved by neutralizing the activation of *TP53* and modulating nuclear factor kappa-light-chain enhancer of activated B cells (*NF-kappaB*) transcriptional activity and expression of apoptotic-related proteins, involving a decrease in BAX protein expression and an increase in BCL2L1 protein expression. On the other hand, the extract did not exert protective effects against tBH-induced apoptosis or reverse the expression of apoptotic-related protein in SK-N-SH-MJD78 cells transfected with a dominant-negative mutant of the inhibitory kappa B-alpha (*IκB-α*) plasmid. The procedure of transfection abolished the potential of the extract to reverse the inhibitory effect of tBH on *NF-kappaB* transcriptional activity. Further, administration of 0.5–1% of extract successfully improved the survival time and locomotor activity in tBH-induced neurotoxicity in ELAV-SCA3tr-Q78 *D. melanogaster* by elevating HSP27 and BCL2L1 protein expression, decreasing mATXN3 protein aggregation, polyQ-expanded mATXN3 and BAX protein expression, and modulating total and phosphorylated TP53 and NF-kappaB protein expression, suggesting amelioration of oxidative stress.

### 10.7. Hyptis sp.

*Hyptis* sp., commonly known as bush mints, belongs to the Lamiaceae family. They are widely distributed in the tropical regions of North and South America and West Africa. In Brazil and India, *Hyptis marrubioides* (L.) Poit. (HM), *Hyptis pectinate* (L.) Poit. (HP), and *Hyptis suaveolens* (L.) Poit. (HS) have been widely used in folk medicine to treat gastrointestinal disorders [137], inflammatory pain of orofacial tissues [138], cramps [139], mosquito-borne diseases [140], and the management of cancer [141] due to their anti-inflammatory and antinociceptive properties. The beneficial effects are attributed to the presence of various bioactive compounds, including rosmarinic acid, essential oils, tannins, saponins, phenols, flavonoids, terpenoids, alkaloids, and sterols [142].

Vilasboas-Campos et al. [95] reported the therapeutic potential of *Hyptis* sp. in an in vivo model of juglone-induced oxidative stress in WT *C. elegans.* The administration of 1 mg/mL of HM, HP, and HS methanol extracts enhanced ARE and increased the survival rate in WT *C. elegans* by scavenging free radicals and upregulating *GSTA4,* which encodes glutathione S-transferase alpha 4, a phase II detoxification enzyme.

Additionally, Vilasboas-Campos et al. [95] also observed an improved motor function assessed by a mechanic stimulus (plate-tap/tap reflex) assay in which vibrations in the agar substrate caused by tapping the petri dish triggered a locomotor response, as well as restoration of the neuronal phenotype following the administration of 1 mg/mL of HS and 0.05–1 mg/mL of HP and HM methanol extracts, from L1 larvae to day 4 after-hatching in a transgenic *C. elegans* model expressing mATXN3. This is caused by an increase in total glutathione and glutathione-disulfide reductase-1 (*GSR-1*) transcriptional activity, contributing to increased intracellular redox potential in promoting survival and eliciting programmed cell death. However, administration of the extracts failed to reverse mATXN3 protein aggregation in the neurons, denoting uncoupling effects between motor performance and mATXN3 protein aggregation.

### 10.8. Paeonia lactiflora Pall.

*Paeonia lactiflora* Pall., commonly known as Bai Shao Yao and garden peony, is a perennial herb belonging to the family Paeoniaceae [96]. It is native to central and eastern Asia. The monoterpene glycosides, such as albiflorin, paeoniflorin, and paeonol, are the major characteristic compounds of its root. Paeoniflorin has been reported to possess various pharmacological effects, such as anti-inflammatory, neuroprotective, sedative, analgesic, anticonvulsant, and antidepressant properties [143], A decoction of the root has been consumed to soothe pain and irritability, nourish blood, alleviate menstrual cramps and muscle spasm, suppress cancer cachexia [144], prevent urothelial carcinoma [145] and manage hepatitis, rheumatoid arthritis, and systemic lupus erythematosus [146].

Chang et al. [96] demonstrated the protective effects of *P. lactiflora* along with its constituents against oxidative stress in in vitro models of ATXN3/Q_75_-GFP 293/SH-SY5Y. The administration of 2–50 µg/mL of aqueous extract, 100 nM of paeoniflorin, and 5 µM of albiflorin, followed by doxycycline and oxaliplatin, reduced mATXN3 aggregation in the cells through upregulation of the heat shock protein 70 (HSP70) family, namely heat shock protein family A (Hsp70) member 8 (*HSPA8)* and heat shock protein family A (Hsp70) member 1A (*HSPA1A*), and heat shock transcription factor 1 (*HSF1*) molecular chaperones. Overexpression of *HSPA1A* also reduced protein aggregation in the transfected cells by modulating the activity of proteins that are involved in cell-cycle machinery, as shown by a low GFP intensity. Chang et al. [96] also observed a decrease in mATXN3 protein aggregation with transient expression of *HSF1* in the cells, suggesting the therapeutic potential of molecular chaperones through an *HSF1*-dependent mechanism in SCA3.

### 10.9. Pueraria lobata (Willd.) Ohwi

*Pueraria lobata* (Willd) Ohwi, commonly known as Ge Gen and kudzu, has been used for the treatment of cardiovascular disease, alcohol intoxication, diabetes [147], stroke [148], and neurodegenerative diseases [149]. The isoflavonoids (puerarin, daidzein, and genistein), saponins, triterpenoids, and phenolic compounds have been found to possess anti-inflammatory [147,150], anti-hypertensive [151], anti-thrombotic [152], chemoprotective, and neuroprotective [97] properties.

Chen et al. [97] reported the protective effects of *P. lobata* and its bioactive compound known as daidzein against oxidative stress activated by carbobenzoxy-L-leucyl-L-leucyl-L-leucinal (MG132, a proteasome inhibitor) in neurons differentiated from iPSCs established from SCA3 patients. The administration of 1 mg/mL of extract or 50 µM of daidzein suppressed the malfunction of UPS induced by MG132 by restoring proteasome activity and decreasing ubiquitination levels in the neurons. Chen et al. [97] also found that the administration of the extract and daidzein restored the levels of MDA and lactate dehydrogenase (LDH), as well as decreased ROS levels. Further, the extract, but not daidzein, reversed an upregulation of caspase-3 activity in the attenuation of apoptosis.

### 10.10. Others

In a study by Chen et al. [98], the administration of individually prepared water extract (0.01–5 mg/mL) from a total of eight herbal remedies, namely *Codonopsis pilosula* (Franch.) Nannf. (Dang Shen), *C. longa*, *Ginkgo biloba* L. (Bai Guo), *Panax ginseng* C.A. Meyer (Ren Shen), *Poria cocos* (Schw.) Wolf (Fu Ling), *P. lobata*, *Rehmannia glutinosa* Libosch. (Di Huang), or *Scrophularia ningpoensis* Hemsl. (Xuan Shen) decreased GFP in MG132-induced oxidative stress in GFPu cells. The attenuation of GFP fluorescent suggests a reversal of the disruption to changes in the cellular redox balance, therefore reestablishing the redox cycle through antioxidant defense mechanisms. Considering the fact that GFPu cells are fluorescent reporters that constantly monitor proteasome activity, an increase in chymotrypsin- and caspase-like proteolytic sites of the 20S proteasome and reduced ubiquitination levels assessed fluorometrically indicate activation of the UPS function, therefore reducing ATXN3/Q_75_ protein aggregation in ATXN3/Q_75_-GFP 293/SH-SY5Y cells.

Further assessment using *C. pilosula*, *G. biloba*, *P. cocos*, *P. lobata,* and *R. glutinosa* has been found to stimulate neuritogenesis in ATXN3/Q_75_-GFP 293/SH-SY5Y cells. The neuroprotective effects were attributed to reduced caspase-3 activity and ubiquitination levels, increased chymotrypsin- and caspase-like proteolytic sites, and downregulation of BAX protein and upregulation of BCL2L1 protein [98].

Successive evaluation employing 10 μg/mL of *P. lobata* and *R. glutinosa* and 0.1 μM of bioactive compounds, namely catalpol, puerarin, and daidzein, identified from *R. glutinosa and P. lobata*, respectively, has been found to decrease GFP and ubiquitination levels in GFPu cells, indicating activation of the UPS function. Additionally, Chen et al. [98] observed that the compounds reduced ATXN3/Q_75_ protein aggregation in ATXN3/Q_75_-GFP 293/SH-SY5Y cells. However, the degradation was not activated by autophagy, as detected by HEK-293 cells expressing red fluorescent protein (DsRed)-LC3-GFP. The herbs and compounds have been found to attenuate enhanced ROS production and increase SOD2 protein expression in ATXN3/Q_75_-GFP 293/SH-SY5Y cells, suggesting improved antioxidant capacity. The compounds also stimulated neuritogenesis in ATXN3/Q_75_-GFP SH-SY5Y cells. The neuroprotective effects were attributed to reduced ATXN3/Q_75_ protein aggregation and caspase-3 activity, increased chymotrypsin- and caspase-like proteolytic sites, and degradation of ubiquitinated proteins, together with downregulation of BAX protein and upregulation of BCL2L1 protein.

**Figure 6:**
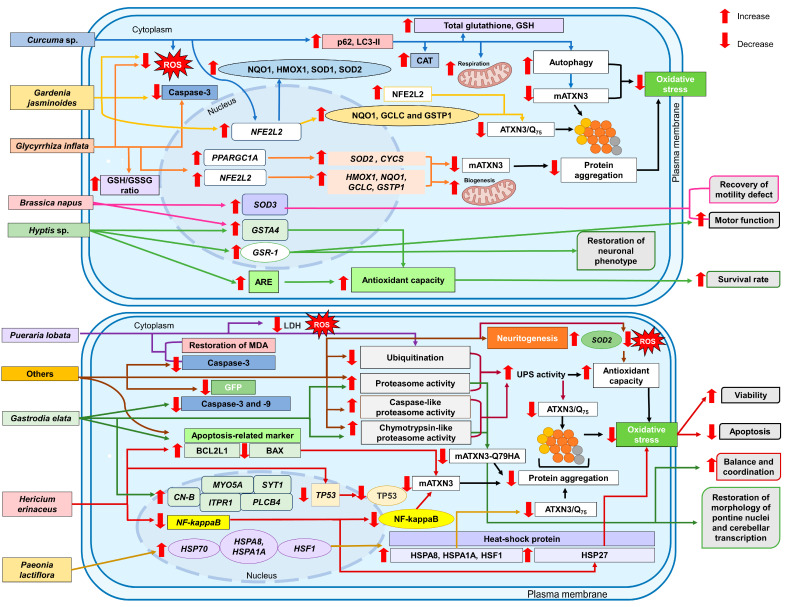
Modes of action of herbal remedies in TCM in the management of SCA3: Activation of antioxidant pathways, activation of UPS and autophagy, regulation of apoptosis, proinflammatory signaling pathway, and chaperones, regulation of mitochondrial function and biogenesis, and restoration of neurotransmission and synaptic plasticity.

Figure 6 summarizes the modes of action exerted by herbal remedies in TCM in the attenuation of oxidative stress in the management of SCA3.

## 11. Limitations and Future Prospects

Despite its widespread use in ethnomedicine, any therapeutic approach that employs a single herb/plant or combination of herbs requires extensive scientific validation and strong pharmacoepidemiologic data to provide evidence of its safety and efficacy [153]. Deeper comprehension of the pharmacokinetics and pharmacodynamic profiles ensures successful future clinical trials. Nonetheless, the major drawback of herbal remedies in TCM is the use of unverified prescriptions. Prescribing decisions should consider the risks and benefits to the individual patient. Allergic and toxic reactions, contraindications, and adverse effects of certain herbal remedies must be confirmed through clinical trials. Therefore, there are still uncertainties pertaining to toxic dose, risks for developmental toxicity, and bioavailability [154,155,156]. Indeed, any form of adverse drug reaction may cause a public health crisis [157].

Further, a formulation containing a mixture of at least two types of herbs in precise proportions [158,159] has been commonly prescribed for treating numerous symptoms, including amnesia, cramping, convulsions, disorientation, insomnia, and unconsciousness, suggesting the association of synergism [160,161]. Consequently, the development of evidence-based TCM and the identification of novel lead compounds and molecular scaffolds are deemed challenging tasks. Combined biological and chemical approaches will foster unprecedented chances for the development of novel formulations of TCM.

Although herbal formulations in TCM have been demonstrated to exert synergistic therapeutic effects [160,161], the safety and effectiveness of these formulations remained questionable due to the lack of well-designed pharmacovigilance studies regarding the formulations. The conventional method commonly used for the characterization of natural products, namely the bioassay-guided fractionation technique, is necessary to isolate and identify bioactive derivatives present in plant extracts [162]. In addition, plant extracts frequently show high viscosity, precipitation, or contain constituents that may activate non-specific protein binding, the presence of non-polar to polar compounds, fluorescence-interfering molecules, as well as metal impurities [163,164,165] that increase the chances of erroneous results. Therefore, interfering substances may need to be removed through column chromatography, droplet counter-current chromatography, and solid-phase extraction. Moreover, molecularly imprinted polymers that possess specialized binding capabilities for targeted analytes can be utilized owing to their thermochemical stability, reusability, and exceptional selectivity and recognition capabilities, enabling the detection of specific target molecules [166].

Further, any consumption of threatened species of herbal remedies could accelerate further demand and lead to overexploitation. Insufficient supply of sustainable raw materials may constitute significant barriers to scientific validation of botanical materials and products. Therefore, a rational equilibrium between cultivation and wild foraging can foster long-term sustainable utilization of TCM resources. A sustainable agriculture approach must be executed to safeguard sustainability and conservation of resources, generate optimal yield and standardized production, as well as ensure the high quality of botanical drug products by minimizing variability in batch-to-batch quality and fulfilling the demands of raw materials required for drug discovery and development research [167].

Excellent choices of preclinical models offer encouraging findings for translation to human clinical trials. For instance, Ritthaphai et al. [168] developed an iPSCs (MUSIi004-A) derived from dermal fibroblasts harboring abnormal CAG repeat expansion in the *ATXN3*. Other than that, the use of mammals, including *D. melanogaster*, transgenic, and knockout mice, is a formidable choice of models due to their highly similar physiological features to hereditary autosomal disease, such as SCA3 [169]. However, there are limitations to using animal models in understanding fundamental aspects, including pathogenesis, genetics, and mechanism. Moreover, predicting treatment effectiveness in clinical trials based on the outcomes of animal studies has been a controversial issue. The limitations of animal-based research have been further underlined by unsuccessful clinical trials established based on animal studies, particularly in rodents [169]. There are currently no models that can accurately mimic the symptoms as well as the cellular mechanisms in SCA3. Despite the usefulness of preclinical studies in providing an in-depth understanding of mechanisms and signaling pathways leading to the discovery of drug targets, validation in human subjects, particularly through randomized controlled trials, is needed to support efficacy claims [170].

Although multiple drugs and herbal remedies in TCM have been proposed as feasible treatments for SCA3 in preclinical studies, interventional approaches involving human subjects are still restricted as most of the early phases of clinical studies are conducted in a small sample size of cohort studies. Further, the reports of SCA3 facts and figures are well documented in Western countries but not in Southeast Asia due to limited genetic testing facilities and coordinated registries [171]. Therefore, collaborative efforts among professionals are necessary to facilitate more diagnosis and develop proper registry systems for the identification of cases and, subsequently, the management of SCA3. In addition, conflicting research findings delay the development of therapeutics for SCA3. Zesiewicz et al. [172] observed that varenicline effectively relieved the disturbances of gait, balance, and posture in SCA3 patients. In contrast, Thomas et al. [173] reported that some patients discontinued the treatment due to intolerable adverse effects such as insomnia, depression, nausea, worsened unsteadiness, and frequent nightmares on top of a lack of benefit. Variances in clinical-trial environment, patient phenotypes, and methods of assessment might contribute to the incompatible results.

Additionally, a disease-modifying therapeutic strategy with gene therapy and gene editing would benefit the advancement of SCA3 in clinical trials. A highly specific approach utilizing short hairpin RNAs (shRNAs) in gene silencing may hold potential for suppressing the mATXN3 protein [174]. The establishment of a safety profile for lentiviral-facilitated delivery of shRNAs could accelerate its translation into clinical studies [175]. Nevertheless, major obstacles have been encountered in the settings of clinical trials, including the absence of shRNAs in target cells, the risk of an inflammatory response against viral particles, the unspecific uptake of shRNAs, and oncogenic effects [176,177,178]. Besides that, the advancement of clustered regularly interspaced short palindromic repeats (CRISPR)/CRISPR-associated endonuclease 9 (Cas9) as a genome editing tool may foster new avenues of study leading to potential treatments for hereditary disorders, including SCA3 [179]. Indeed, CRISPR has been revealed to remove CAG repeats in iPSCs derived from an SCA3 patient [180]. However, off-target effects of CRISPR/Cas9 may result in untargeted genomic sites [181].

## 12. Conclusions

In conclusion, this systematic review provides a broad and optimistic understanding toward the therapeutic potential of herbal remedies in TCM for mitigating oxidative stress in SCA3. The modes of action contributing to the attenuation of oxidative stress are activation of antioxidant pathways, ubiquitin–proteasome system and autophagy, regulation of apoptosis, proinflammatory signaling pathway and chaperones, regulation of mitochondrial function and biogenesis, and restoration of neurotransmission and synaptic plasticity. However, extensive preclinical evidence and a safety profile assessment are necessary prior to its translation into clinical practice.

## Figures and Tables

**Table 1:** Herbal remedies in TCM for the attenuation of oxidative stress in SCA3.

Herbal Remedy	Model	Dose	Finding	Mode of Action	Ref.
*Brassica napus* L.	Transgenic *C. elegans* expressing mATXN3	0.1–5 mg/mL of ethanol extract. 0.001–1 mg/mL of sinapine.	Restoration of motor function. Recovery of motility defect.	↑ *GSTA4* and *SOD3* mRNA expression.	[92]
*Curcuma* sp.	SK-N-SH-MJD78 cells	0.3–5 µM of JM17	↑ Mitochondrial respiration. ↓ mATXN3 protein and its aggregation. ↑ Degradation of mATXN3 through autophagy. ↓ ROS level. ↑ NQO1, HMOX1, SOD1, and SOD2 protein expression. ↑ CAT level, total glutathione, and GSH levels. ↓ Oxidative stress.	↑ p62 and LC3-II protein expression. ↑ *NFE2L2* mRNA expression.	[68]
*Gardenia jasminoides* Ellis	HEK-293 cells with inducible ATXN3/Q_75_-GFP expression. Flp-In SH-SY5Y cells with inducible ATXN3/Q_14–75_-GFP expression.	1–10 µg/mL of aqueous extract. 50–500 nM of genipin. 500 nM of geniposide. 100 nM of crocin.	↓ ATXN3/Q_75_ protein aggregation. ↓ ROS level.	↑ NFE2L2 protein expression. ↑ NQO1, GCLC, and GSTP1 protein expression. ↓ caspase-3 activity.	[56]
*Gastrodia elata* Blume	Transgenic mice expressing mATXN3-Q79HA	5–50 mg/kg of T1-11. 5–50 mg/kg of JMF1907.	↑ Proteasome activity. ↑ Chymotrypsin-like proteasome activity. ↓ mATXN3-Q79HA protein. ↑ Balance and coordination. Restoration of morphology of pontine nuclei and cerebellar transcription.	↓ BAX protein expression. ↑ BCL2L1 protein expression. ↓ caspase-3 and -9 activities. Restoration of *MYO5A*, *SYT1*, *CN-B*, *ITPR1,* and *PLCB4* mRNA expression.	[54]
*Glycyrrhiza inflata* Batalin	HEK-293 cells with inducible ATXN3/Q_75_-GFP expression. Flp-In SH-SY5Y cells with inducible ATXN3/Q_14–75_-GFP expression.	5–5000 µg/mL of aqueous extract. 0.1–100 µM of AMGZ. 1–1000 nM of licochalcone A.	↓ mATXN3 protein aggregation. ↑ Mitochondrial biogenesis. ↑ GSH/GSSG ratio. ↓ ROS level. ↓ Oxidative stress.	↑ *PPARGC1A*, *SOD2,* and *CYCS* mRNA expression. ↑ *NFE2L2*, *HMOX1*, *NQO1*, *GCLC*, and *GSTP1* mRNA expression. ↓ caspase-3 activity.	[93]
*Hericium erinaceus* (Bull.: Fr.) Pers.	tBH-induced oxidative stress in SK-N-SH-MJD78 cells.	1.25–5 μg/mL of ethanol extract.	↑ Viability. ↓ Apoptosis. ↓ mATXN3 protein and its aggregation.	↓ TP53 and NF-kappaB mRNA and protein expression. ↓ BAX protein expression. ↑ BCL2L1 and HSP27 protein expression.	[94]
	tBH-induced oxidative stress in ELAV-SCA3tr-Q78 *D. melanogaster*.	0.5–1% of ethanol extract.	↑ Survival time and locomotor activity. ↓ Oxidative stress. ↓ mATXN3 protein and its aggregation.		
*Hyptis* sp.	Juglone-induced oxidative stress in WT *C. elegans*.	0.05–1 mg/mL of methanol extract.	↑ ARE. ↑ Survival rate.	↑ Antioxidant capacity. ↑ *GSTA4* mRNA expression.	[95]
	Transgenic *C. elegans* expressing mATXN3.		↑ Motor function. Restoration of neuronal phenotype.	↑ Total glutathione and *GSR-1* mRNA expression.	
*Paeonia lactiflora* Pall.	HEK-293 cells with inducible ATXN3/Q_75_-GFP expression.	2–50 µg/mL of aqueous extract. 100 nM of paeoniflorin. 5 µM of albiflorin.	↓ ATXN3/Q_75_ protein aggregation.	↑ *HSF1*, *HSPA8*, *HSP70*, and *HSPA1A* mRNA expression.	[96]
	Flp-In SH-SY5Y cells with inducible ATXN3/Q_75_-GFP expression.			↑ HSF1, HSPA8, and HSPA1A protein expression.	
*Pueraria lobata* (Willd.) Ohwi	MG132-induced oxidative stress in iPSCs-derived neurons.	1 mg/mL of the extract. 50 µM of daidzein.	↑ UPS and proteasome activities. ↓ Ubiquitination. Restoration of MDA, LDH, and ROS levels.	↓ caspase-3 activity.	[97]
Other *Codonopsis pilosula* (Franch.) Nannf. *Curcuma longa* L. *Ginkgo biloba* L. *Panax ginseng* C.A. Meyer. *Poria cocos* (Schw.) Wolf. *P. lobata*. *Rehmannia glutinosa* Libosch. *Scrophularia ningpoensis* Hemsl.	MG132-induced oxidative stress in GFPu reporter cells.	0.01–5 mg/mL of water extract.	↓ GFP. ↑ Chymotrypsin- and caspase-like proteasome activity. ↓ Ubiquitination. ↑ UPS and proteasome activities.	↑ Antioxidant capacity.	[98]
	HEK-293 and SH-SY5Y cells with inducible ATXN3/Q_75_-GFP expression.		↓ ATXN3/Q_75_ protein aggregation.		
*C. pilosula*. *G. biloba*. *P. cocos*. *P. lobata*. *R. glutinosa*.	HEK-293 and SH-SY5Y cells with inducible ATXN3/Q_75_-GFP expression.		Stimulation of neuritogenesis. ↓ Ubiquitination. ↑ Chymotrypsin- and caspase-like proteasome activity.	↓ caspase-3 activity. ↓ BAX protein expression. ↑ BCL2L1 protein expression.	
*P. lobata*. *R. glutinosa*.	MG132-induced oxidative stress in GFPu reporter cells.	0.01–5 mg/mL of water extract. 0.1 µM of catalpol, puerarin, and daidzein.	↓ GFP. ↓ Ubiquitination. ↑ UPS and proteasome activities.	↑ Antioxidant capacity.	
	HEK-293 and SH-SY5Y cells with inducible ATXN3/Q_75_-GFP expression.		↓ ATXN3/Q_75_ protein aggregation. ↓ ROS level.	↑ SOD2 protein expression.	
	SH-SY5Y cells with inducible ATXN3/Q_75_-GFP expression.		Stimulation of neuritogenesis. ↓ ATXN3/Q_75_ protein aggregation. ↓ Ubiquitination. ↑ Chymotrypsin- and caspase-like proteasome activity.	↓ caspase-3 activity ↓ BAX protein expression. ↑ BCL2L1 protein expression.	

↑: increase; ↓: decrease.

## Data Availability

Not applicable.

## Abbreviations

AD, Alzheimer’s disease; AKT, protein kinase B; AMGZ, ammonium glycyrrhizinate; AMPK, 5′-adenosine monophosphate (*AMP*)-activated protein kinase; ARE, antioxidant response element; ATM, ataxia-telangiectasia mutated; ATP, adenosine 5′-triphosphate; ATR, ATM-Rad3-related; ATXN3, ataxin-3; ATXN-3-Q79HA, hemagglutinin (HA)-tagged polyglutamine-expanded ataxin-3-Q79; BAX, BCL2 associated X, apoptosis regulator; BCL2, B-cell lymphoma 2; BCL2L1, BCL2 like 1; CAG, cytosine-adenine-guanidine; Cas9, CRISPR-associated endonuclease 9; CAT, catalase; CHEK1, checkpoint kinase 1; CN-B, calcineurin subunit B; CoQ10, coenzyme Q10; COS-7, African green monkey kidney fibroblast-like; CRISPR, clustered regularly interspaced short palindromic repeats; CYCS, cytochrome c, somatic; DDR, DNA damage response; DMF, dimethyl fumarate; DNA, deoxyribonucleic acid; DsRed, red fluorescent protein; Flp, flippase; FOXO, forkhead box protein O; FOXO4, forkhead box O4; GCLC, glutamate-cysteine ligase catalytic subunit; GFP, green fluorescent protein; GPx, glutathione peroxidase; GR, glutathione reductase; GSH, reduced glutathione; GSR-1, glutathione-disulfide reductase 1; GSSG, oxidized glutathione or glutathione disulfide; GSTA4, glutathione S-transferase alpha 4; GSTP1, glutathione S-transferase pi 1; H_2_O_2_, hydrogen peroxide; HA, hemagglutinin; HEK-293, human embryonic kidney 293; HeLa, human adenocarcinoma; HMOX1, heme oxygenase 1; HO^•^, hydroxyl radical; HSF1, heat shock transcription factor 1; HSP, heat shock protein; HSP27, heat shock 27kD protein 1; HSP70, heat shock protein 70 family; HSPA1A, heat shock protein family A (Hsp70) member 1A; HSPA8, heat shock protein family A (Hsp70) member 8; HTS, High throughput screening; IκB-α, inhibitory-kappa B-alpha; iPSC, induced pluripotent stem cell; ITPR1, inositol 1,4,5-trisphosphate receptor type 1; JM17, curcumin analog; JMF1907, N6-(3-indolylethyl) adenosine; LC3, microtubule-associated protein light chain 3; LDH, lactate dehydrogenase; mATXN3, mutant ATXN3; MDA, malondialdehyde; MDC1, mediator of DNA damage checkpoint 1; MFN1, mitofusin 1; MFN2, mitofusin 2; MG132, carbobenzoxy-L-leucyl-L-leucyl-L-leucinal; MMP, mitochondrial membrane potential; mPTP, mitochondrial permeability transition pore; mRNA, messenger ribonucleic acid; mtDNA, mitochondrial DNA; mTOR, mechanistic target of rapamycin kinase; MYO5A, myosin VA; NADPH, NADPH oxidase 1; NF-kappaB, nuclear factor kappa-light-chain-enhancer of activated B cells; NFE2L2, nuclear factor (erythroid-derived 2) like bZIP transcription factor 2; NQO1, NAD(P)H quinone dehydrogenase 1; O_2_^•−^, superoxide anion; p62, nucleoporin 62; PD, Parkinson’s disease; PI3K, phosphoinositide-3-kinase; PINK1, PTEN-induced kinase 1; PLCB4, phospholipase C beta 4; PMAIP1, phorbol-12-myristate-13-acetate-induced protein 1; PNKP, polynucleotide kinase 3′-phosphatase; polyQ, polyglutamine; PPARGC1A, peroxisome proliferative activated receptor, gamma, coactivator 1; PRISMA, Preferred Reporting Items for Systematic Review and Meta-Analyses; PROSPERO, The International Prospective Register of Systematic Reviews; RNA, ribonucleic acid; ROS, reactive oxygen species; RSP, rapeseed pomace; RTA408, omaveloxolone; SARA, Scale for Assessment and Rating of Ataxia; SCA3, spinocerebellar ataxia type 3; shRNAs, short hairpin RNAs; SH-SY5Y, human neuroblastoma; SK-N-SH, human neuroblastoma; Smac, second mitochondria-derived activator of caspase; SOD, superoxide dismutase; SOD1, superoxide dismutase 1; SOD2, superoxide dismutase 2; SOD3, superoxide dismutase 3; SYT1, synaptotagmin 1; T1-11, N6-(4-hydroxybenzyl) adenosine; tBH, tert-butyl hydroperoxide; TCM, traditional Chinese medicine; TFEB, transcription factor EB; TP53, tumor protein 53; UHDRS, Unified Huntington’s Disease Rating Scale; ULK, unc-51-like autophagy activating kinase; UPS, ubiquitin–proteasome system; VDAC1, voltage-dependent anion channel 1; WT, wild-type.
